# Methacrylated Cartilage ECM-Based Hydrogels as Injectables and Bioinks for Cartilage Tissue Engineering

**DOI:** 10.3390/biom12020216

**Published:** 2022-01-27

**Authors:** Kevin Behan, Alexandre Dufour, Orquidea Garcia, Daniel Kelly

**Affiliations:** 1 Trinity Centre for Biomedical Engineering, Trinity Biomedical Sciences Institute, Trinity College Dublin, D02 PN40 Dublin, Ireland; behank@tcd.ie (K.B.); alexandre.dufour3@univ-lyon1.fr (A.D.); 2 Department of Mechanical, Manufacturing and Biomedical Engineering, School of Engineering, Trinity College Dublin, D02 PN40 Dublin, Ireland; 3 Johnson & Johnson Services, Inc., 31 Technology Drive, Irvine, CA 92618, USA; ogarci22@ITS.JNJ.com; 4 Advanced Materials and Bioengineering Research Centre (AMBER), CRANN, Naughton Institute, Trinity College Dublin, D02 PN40 Dublin, Ireland; 5 Department of Anatomy, Royal College of Surgeons in Ireland, D02 YN77 Dublin, Ireland

**Keywords:** bioinks, cartilage, bioprinting

## Abstract

Articular cartilage (AC) possesses a limited healing potential, meaning that untreated focal joint defects typically progress, leading to the development of degenerative diseases such as osteoarthritis. Several clinical strategies exist that aim to regenerate AC; however, recapitulation of a fully functional, load-bearing tissue remains a significant challenge. This can be attributed, at least in part, to a paucity of biomaterials that truly mimic the native tissue and provide appropriate cues to direct its regeneration. The main structural component of articular cartilage, type II collagen, does not readily gelate at body temperature, challenging the development of cartilage extracellular matrix (cECM)-derived injectable hydrogels and bioinks for AC tissue engineering and bioprinting applications. Here, we describe the development and rheological characterisation of a methacrylated cartilage ECM-based hydrogel/bioink (cECM-MA), which could be photocrosslinked when exposed to ultraviolet (UV) light. Functionalisation of the collagen backbone with methacryloyl groups had a negligible effect on triple helix stability, as demonstrated by circular dichroism spectroscopy. These cECM-MA bioinks demonstrated shear-thinning properties and could be loaded with bone marrow mesenchymal stem cells (BM-MSCs), micro-extruded to generate self-supporting 3D constructs of predefined size and shape, and then photocrosslinked using UV light. Analysis of the cell-laden constructs showed that the BM-MSCs were viable post-printing and underwent chondrogenesis in vitro, generating a tissue rich in sulphated glycosaminoglycans and collagens. These results support the use of methacrylated, tissue-specific ECM-derived hydrogels as bioinks for 3D bioprinting and/or as injectables for cartilage tissue engineering applications.

## 1. Introduction

Articular cartilage (AC) is a highly specialised connective tissue found in diarthrodial joints, the function of which is to enable friction-reduced movement and distribute mechanical loads across the joint surface. The extracellular matrix (ECM) of AC is predominately composed of proteoglycans and type II collagen. The specific arrangement of the ECM components in AC provides the tissue with unique biomechanical properties that allow the tissue to function over many decades. However, the relatively low cell density coupled with avascularity results in a tissue with negligible healing potential [1]. Existing clinical strategies that aim to regenerate cartilage tissue fail to recapitulate the composition and structural complexity of the native tissue [2,3], resulting in suboptimal mechanical properties and the eventual failure of the repair. This has motivated increased interest in the development of biomimetic scaffolds and hydrogels [4,5,6], as well as novel manufacturing methods, such as 3D bioprinting [7,8], to assist in the engineering of structurally complex tissues, such as AC. A central challenge in these efforts is the development of chondro-inductive biomaterials that mimic key aspects of the structure and composition of the native tissue.

Decellularised ECM-derived scaffolds and hydrogels are well established in the fields of tissue engineering and regenerative medicine applications [9,10,11], and such biomaterials are now in widespread clinical use. Despite this, the ideal source of ECM for the engineering of specific tissues and organs remains an active area of research. In the case of musculoskeletal tissues, it has been demonstrated that decellularised tendon ECM can promote the tendon-specific differentiation of mesenchymal stem/stromal cells (MSCs) [12], and meniscus ECM supports a fibrocartilage phenotype [13,14], while articular cartilage ECM has been shown to promote chondrogenesis. This has increased the use of different tissue-specific ECMs in the development of bioinks for 3D printing applications [15], with, for example, the addition of solubilised cartilage [16] or meniscus [17] ECM, shown to promote a cartilage or meniscus phenotype, respectively. A limitation of many previous approaches is that the ECM is only a minor component of the overall bioink, potentially limiting its bioactivity. This motivates the development of injectable hydrogels and bioinks derived primarily or exclusively from the ECM of a specific tissue of interest. 

While numerous different strategies are available for the crosslinking of hydrogels [18,19], light-based approaches are particularly attractive for tissue engineering and bioprinting applications. The overall goal of this study was to develop a photocrosslinkable cartilage ECM-derived hydrogel for use either as an injectable or as a bioink for cartilage tissue engineering applications. The major component of mature articular cartilage is type II collagen, which is characterised by its poor thermal gelation when compared with type I collagen [20]. Yang et al. recently reported the synthesis of a methacrylated type II collagen (mono-component), which can be photocrosslinked to produce stable hydrogels [21]. Encapsulated MSCs within such hydrogels demonstrated excellent viability and were able to condensate and undergo chondrogenesis. Here, we sought to build upon this work to develop a methacrylated cartilage ECM-based hydrogel/bioink (cECM-MA) that is rich in type II collagen. We further sought to characterise the rheology of such hydrogels, their suitability for 3D bioprinting applications, and their capacity to support the differentiation of bone marrow-derived MSCs. 

## 2. Materials and Methods

### 2.1. Isolation and Solubilisation of Porcine Knee Cartilage

Porcine AC was harvested from the femoral condyle and trochlear ridge of 3–4-month-old female pigs (Danish Duroc). The AC was shaved using a biopsy punch; shavings were diced and then stored in PBS at 4 °C. AC was solubilised as per the method described by Browe et al. [22]. Briefly, diced AC was pre-treated with a 0.2 mol L^−1^ solution of sodium hydroxide to remove various sulphated glycosaminoglycans (sGAGs) from the ECM, which were separated by centrifugation. The pellet was washed with ultra-pure (UP) water until close to neutral pH and then dissolved in 0.5 mol L^−1^ acetic acid. The dissolved matrix (predominately collagen) was precipitated by salting out with 5 mol L^−1^ sodium chloride, redissolved in acetic acid, and precipitated again. The solution was dialysed against 0.02 mol L^−1^ dibasic sodium hydrogen phosphate, and the dialysate was freeze dried for 48 h. As required, the ECM pellet was processed for methacrylation, as detailed below.

### 2.2. Methacrylation of cECM

**Scheme 1:**
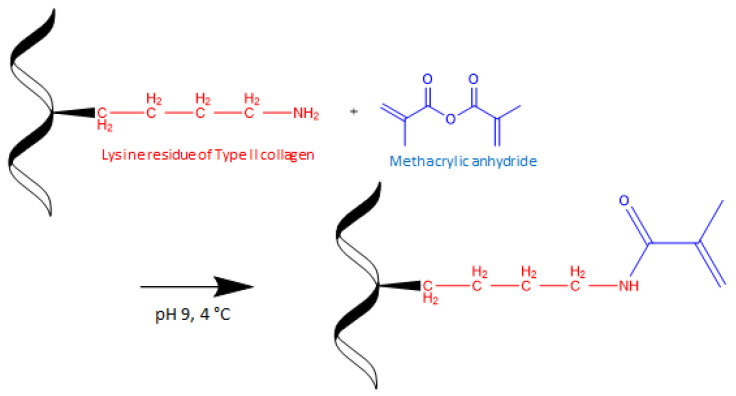
Methacrylation of cECM.

The methacrylation protocol was adapted from [23]. The specific reaction conditions were chosen so that the cECM collagens were fully functionalised. After washing with UP water, the pellet was dissolved in 0.25 mol L^−1^ carbonate–bicarbonate (CB) buffer (~pH 9). This pH deprotonates the residual lysines on collagen triple helix, increasing reactivity towards methacrylic anhydride (MAA), while also minimising hydrolysis of the anhydride to methacrylic acid. The final concentration of ECM in CB buffer was 0.25% (*w*/*v*). MAA was added dropwise (2.5% *v*/*v*) to the solution, keeping the temperature at 4 °C (Scheme 1). The reaction was carried out for 3 h and then transferred to a dialysis membrane (SpectraPor, 12–14 kDa) for 48 h (0.02 mol L^−1^ dibasic sodium hydrogen phosphate; effective dilution factor (df) of 25). The dialysis buffer was changed after 24 h (effective df of 625). Dialysate was frozen at −20 °C and freeze dried at −10 °C for 48 h, including secondary drying at 20 °C for 2 h. The final product was stored at −20 °C and protected from light. Methacrylation of ECM collagens was confirmed by ^1^H-nuclear magnetic spectroscopy; 10 mg of cECM-MA was dissolved in 0.7 mL of deuterated water (acidified with 10 µL of deuterated acetic acid, CDCOOD) and transferred to an NMR analysis tube. The analysis was performed using an Agilent 400 MR equipped with a 5 mm OneNMR probe for proton and multinuclear detection.

### 2.3. Formulation of Articular Cartilage Extracellular Matrix Methacrylate (cECM-MA) Bioinks and Printability

cECM-MA was weighed according to the final concentration of cECM-MA in the bioink (1 and 2% (*w*/*v*)) and combined with gelatin (3.5%) as viscosity modifier. The weighed material was carefully transferred to an empty 3 mL syringe barrel and connected via Luer lock to another syringe containing hgDMEM (700 µL). The cECM-MA was then wetted with the hgDMEM and slowly passed back and forth between the two syringes, allowing mixing and gradual dissolution. The hgDMEM turned pink due to the slightly alkaline pH of dialysed cECM-MA. LAP photoinitiator stock (100 μL) resuspended in hgDMEM was added to give a final concentration of 0.25%. Bioink printability was assessed according to the spreading ratio. Bioinks were extruded, and the spreading ratio was calculated from measurements of the printed filaments using ImageJ software. Fidelity of multi-layer constructs was assessed by comparing print and file dimensions.

### 2.4. Rheological Analysis of cECM-MA Bioinks

Bioink rheological properties were assessed using an MCR 301 rheometer (Anton Paar GmbH, Graz, Austria). The viscosity of the bioinks was measured against shear rate, allowing determination of yield stress, flow index, and consistency index. To model the extrusion process, recovery of the viscosity from high to low shear (895 to 0.1/s) was measured. Viscoelastic properties of unmodified cECM (G′, G′) at three different concentrations were measured as a function of strain and frequency. Photocrosslinking kinetics of formulations at 1 and 2% cECM-MA, with and without gelatin (3.5%), were studied by measuring G′ and G″ before and after exposure to light. 

### 2.5. Cell Isolation and Expansion 

Bone marrow-derived mesenchymal stem cells (BM-MSCs) were isolated from the sternum of a goat donor (Saanen, female, approximately 4 years old). The marrow was collected with a surgical curette; cut into small chunks; diluted in expansion medium containing high-glucose Dulbecco’s modified Eagle medium (hgDMEM) (Biosciences), supplemented with 10% foetal bovine serum (FBS) and penicillin (100 U/mL)–streptomycin (100 μg/mL) (all from Bioscience); and vortexed for 5 min to liberate the cells. The medium was collected, and the procedure was repeated until the marrow was visibly whiter than before vortexing. The cells were centrifuged at 650× *g* for 5 min, resuspended in fresh expansion medium twice, then triturated with a 16G needle until a homogenous mixture was obtained, and filtered through a 40 μm cell sieve (Sarstedt). Cell counting was performed with trypan blue in the presence of acetic acid (3% final) before plating at a density of 3 × 10^5^ cells/cm^2^. Following colony formation, cells were trypsinised, counted, and re-plated for 2 additional passages at a density of 5 × 10^3^ cells/cm^2^ at 5% pO_2_ in expansion media supplemented with 5 ng/mL of fibroblast growth factor (FGF)-2 (PeproTech Ltd., London, UK), which was changed three times per week.

### 2.6. Cell Culture in cECM-MA Hydrogels

To prepare 1 mL of cell–gel suspension, cECM-MA and gelatin (type A, Sigma-Aldrich, Arklow, Ireland) were dissolved in 700 µL of hgDMEM at 37 °C, followed by the addition of 100 µL of 2.5% (*w*/*v*). Lithium phenyl-2,4,6-trimethyl-benzoyl phosphinate (LAP) (Sigma-Aldrich, Arklow, Ireland) and 200 µL of cells were resuspended in hgDMEM. The final volume was then adjusted to 1 mL with hgDMEM so that the final concentrations were 1 or 2% (*w*/*v*) cECM-MA, 3.5% (*w*/*v*) gelatin, 0.25% (*w*/*v*) LAP, and 20 × 10^6^ cells/mL. The gel–cell mixture was then poured in 5 mm × 3 mm cylindrical moulds 3D printed with dental resin (Formlabs) and exposed to UV light for 10 min. The cell-embedded gels were finally transferred to a 24-well plate and cultured in chondrogenic media consisting of hgDMEM GlutaMAX supplemented with penicillin (100 U/mL)–streptomycin (100 μg/mL), 100 µg/mL sodium pyruvate, 40 µg/mL L-proline, 50 µg/mL L-ascorbic acid-2-phosphate, 4.7 µg/mL linoleic acid, 1.5 mg/mL bovine serum albumin (BSA), 1X insulin–transferrin–selenium, 100 nmol L^−1^ dexamethasone (all from Sigma-Aldrich), and 10 ng/mL human transforming growth factor-beta (TGF-b) (PeproTech Ltd., London, UK). Cells were maintained at 5% pO_2_ for 21 days, and the chondrogenic medium was changed every two days.

### 2.7. Cell Viability

Cell viability was evaluated 7 days following gel embedding using a Live/Dead assay. Cell-seeded gels were rinsed in phosphate-buffered saline (PBS) and incubated in a solution containing 4 μmol L^−1^ ethidium homodimer-1 and 2 μ mol L^−1^ calcein (Cambridge Biosciences, Cambridge, UK) for 30 min. Following incubation, the scaffolds were rinsed again and imaged with an Olympus BX51 upright microscope equipped with an LED light source (CoolLED, Andover, UK) and an Olympus DP73 digital camera.

### 2.8. Biochemical Analyses

After 21 days of in vitro culture, constructs were washed in PBS and frozen for subsequent assessment. Each construct was digested with papain (3.88 U/mL) in 100 mM sodium phosphate-5 mM ethylenediaminetetraacetic acid (EDTA) buffer (pH 6.5) with 10 mmol L^−1^ L-cysteine–HCl (all from Sigma–Aldrich) at 60 °C and 10 rpm for 18 h. DNA content was quantified using the Hoechst Bisbenzimide 33,258 dye assay, with a calf thymus DNA standard, and the amount of sGAGs was quantified using the dimethyl methylene blue dye-binding assay (DMMB) (Blyscan, Biocolor Ltd., Carrickfergus, UK), with a chondroitin sulphate standard.

### 2.9. Histological and Immunohistochemical Analyses

Engineered tissue constructs were fixed in 4% paraformaldehyde, dehydrated in a graded series of ethanols, embedded in paraffin wax, and sectioned at 5 µm. Deparaffinised sections were stained with Goldner’s Masson trichrome. For detection of sGAGs, sections were stained with safranin O/fast green.

Collagen types I and II were also evaluated using a standard immunohistochemical technique. Rehydrated sections were treated with pronase (32 PUK/mL, Sigma-Aldrich) at 37 °C for 5 min and then incubated in blocking buffer containing 1% BSA and 10% goat serum (Sigma, Arklow, Ireland) in 1Xx PBS for 1 h at room temperature (RT). Tissue sections were then incubated with anti-type I (Abcam, ref. 90395, mouse monoclonal IgG, 1:400) or anti-type II collagen (Abcam, ref. 3092, mouse monoclonal IgG, 1:100) primary antibody diluted in blocking buffer overnight at 4 °C in a humidified chamber. Samples were incubated with 3% hydrogen peroxide solution (Sigma-Aldrich) for 20 min to block endo-peroxidase activity and then with secondary antibody (Sigma-Aldrich, ref. B7151, anti-Mouse IgG) diluted in blocking solution (1.5:200 for detection of type I collagen and 1:300 for detection of type II collagen) for 1 h at RT. Following a 45 min incubation period with ABC reagent (ABC Elite kit Vectastain PK-400, Vector Labs, Burlingame, CA, USA), the DAB substrate (SK-4100, Vector Labs) was added to the tissue sections and left until a brown staining was observed in the positive control. Histological and immunohistochemical samples were imaged with a slide scanner (Scanscope, Leica biosystems, Wetzlar, Germany) and analysed with Aperio software (Leica biosystems).

### 2.10. Mechanical Testing

Photocrosslinked cECM-MA hydrogels (Փ5 mm × h 3 mm, *n* = 5) were subjected to a uniaxial unconfined compression test in PBS using a single-column mechanical tester (Zwick/Roell Z2.5, Herefordshire, UK) with a 5N load cell. Briefly, constructs were kept hydrated in PBS bath maintained at room temperature. A preload of 0.01 N was applied for 60 s to ensure that top and bottom construct surfaces were in direct contact with the impermeable loading platens. Force was zeroed after preload and then followed by a cyclic compression test consisting of compressive cycles with increasing strain amplitude of 10, 20, and 30% in sequence at a rate of 2.22% strain per second. A holding time of 10 s between each cycle was added to allow full height recovery of the samples. The load versus displacement data were recorded throughout. The apparent stress and strain were calculated by dividing the load value with the initial crosssectional area of each sample and the displacement value with the initial sample height, respectively. Cumulative percentage of the total height loss and height loss per cycle was calculated to characterise the super-elastic properties of the constructs. Scaffold permanent deformation (PD) at each cycle was calculated as follows: PD = (Test Speed*Δ*tn*)/*h*0, where Δ*tn* is the interval of time at the start of the nth cycle in which no force is applied to the sample, and *h*0 is the initial height of the sample. 

### 2.11. Statistical Analysis

Statistical analysis was performed with GraphPad (GraphPad Software, La Jolla, CA, USA). Experimental groups in the rheological tests were analysed for significance using two-way ANOVA. Experimental groups in the biochemical tests were analysed for significance using the nonparametric Mann–Whitney test (accepted for *p* ≤ 0.05).

## 3. Results and Discussion

### 3.1. Rheological Properties of Solubilised cECM

**Figure 1:**
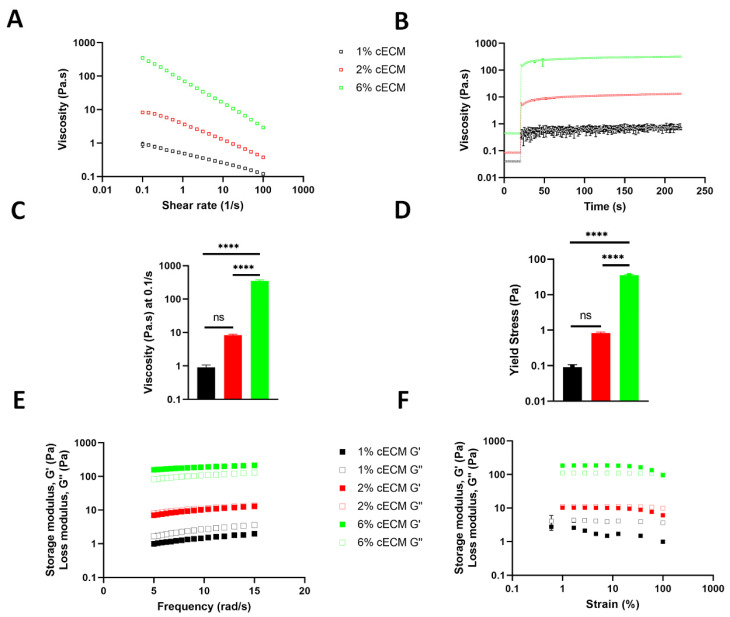
Rheological properties of formulations containing unmodified cartilage ECM (*n* = 3). (**A**) Shear rate sweep from 0.1 to 100/s. (**B**) Shear recovery from 895/s (first 20 s) to 0.1/s (last 200 s). (**C**) Effect of cECM concentration on the viscosity at a shear rate of 0.1/s (close to zero shear) (ns = not significant; **** = *p* < 0.0001). (**D**) Effect of cECM concentration on the yield stress (flow initiation) (ns = not significant; **** = *p* < 0.0001). (**E**) Frequency sweeps of 1, 2, and 6% cECM. (**F**) Amplitude sweeps of 1, 2, and 6% cECM.

Collagen-based hydrogels are used extensively in 3D cell culture and 3D bioprinting. Type I collagen (Coll-I) without chemical modification can form physically crosslinked hydrogels at concentrations as low as 0.5% *w/v* at temperatures as low as 25 °C [23]. In contrast, type II collagen (Coll-II) generally undergoes poor gelation in the physiological temperature regime [24] and is commonly used in conjunction with Coll-I or other polymers to form stable hydrogels [25]. Solubilised cartilage ECM that has been pre-treated to remove sGAGs, with subsequent enzymatic solubilisation of the collagen network, can be considered as a solution consisting predominantly of Coll-II monomers, with the collagen network of articular cartilage making up between 50 and 70% of the tissue dry weight [9]. To assess the potential printability of unmodified solubilised cECM, the material was formulated at different concentrations, and their rheological properties were assessed. Viscosity measurements provide an insight into how these formulations might behave during injection and/or 3D printing. For a given shear rate, the viscosity increased as the concentration of the cECM went from 1 to 6% (Figure 1A). This may be due to the Coll-II triple helix monomers coming into closer contact at higher cECM concentrations, allowing reorganisation of monomers via supramolecular interactions. Rheologically, this can be thought of as the emergence of structure, providing additional resistance to flow. The cECM also displayed shear-thinning behaviour at all concentrations.

The effect of the cECM concentration on shear recovery was assessed (Figure 1B). At a shear rate of 895 Pa/s, the viscosity dropped considerably due to deformation of the sample components and subsequent flow. Viscosity recovered when the shear rate decreased to 0.1 Pa/s. The storage modulus (G′) and loss modulus (G″) were calculated as a function of frequency (Figure 1E) and applied strain (Figure 1F). G′ increased by approximately 40-fold when going from 1 to 6% cECM (Figure 1E,F). These results suggest improved printability at higher concentrations of cECM, but they also demonstrate that elastic gels only form at higher concentrations, pointing to the need for further functionalisation of the cECM to generate useful bioinks and/or stable constructs for tissue engineering applications. 

### 3.2. Collagen Functionalisation

**Figure 2:**
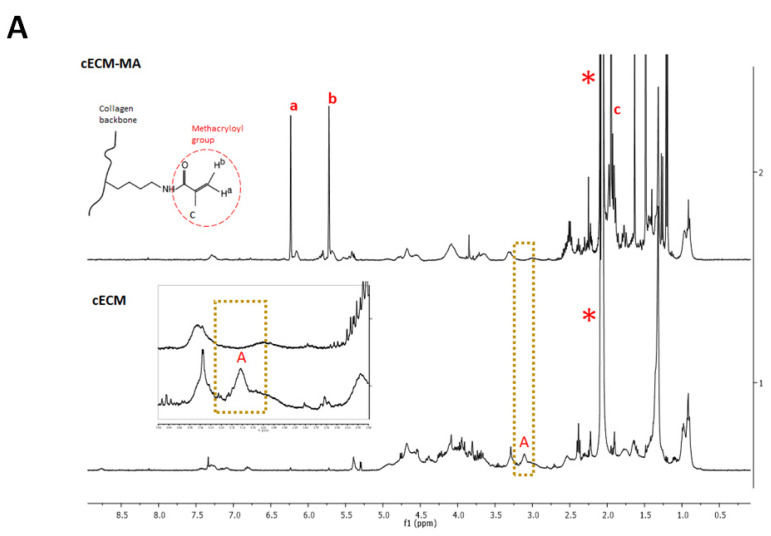

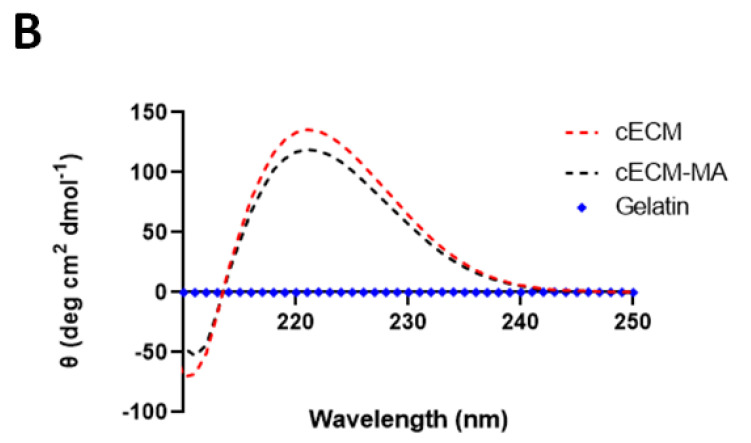
(**A**) ^1^H-NMR spectra of cartilage ECM (cECM) and cartilage ECM methacryloyl (cECM-MA) recorded in CDCOOD (*) at room temperature. (**B**) CD spectra of cECM, cECM-MA, and gelatin.

Several examples in the literature have shown that collagen can be functionalised into a photopolymerisable macromer, capable of crosslinking into a 3D network [25,26]. cECM in solution was reacted with methacrylic anhydride, providing methacryloyl functionality. The reaction of lysine residues on collagen with methacrylic anhydride was elucidated by ^1^H-NMR (Figure 2A). The peaks a (6.2 ppm), b (5.7 ppm), and c (1.9 ppm) demonstrate the incorporation of double bonds onto the collagen triple helix and associated random coils. The a and b peaks represent the alkenyl hydrogen resonances, with the methyl group being represented by peak c. The degree of functionalisation can be estimated from the integrated intensity A (3.2 ppm) representing the epsilon protons of the lysine residues. The disappearance of peak A suggests that the lysine residues were fully reacted. The stability of the collagen triple helix, pre- and post-methacrylation, was confirmed by circular dichroism (Figure 1B). Free triple helix appears as the positive ellipticity band at 220 nm, while random coils appear as a negative ellipticity band at 210 nm [27]. Gelatin type A is a denatured form of collagen that has subsequently undergone hydrolysis, resulting in a lower molecular weight species that display no circular dichroism. Interactions specific to the collagen triple helix may provide increased bioactivity to cECM hydrogels.

### 3.3. Rheological Properties of cECM-MA-Based Inks

**Figure 3:**
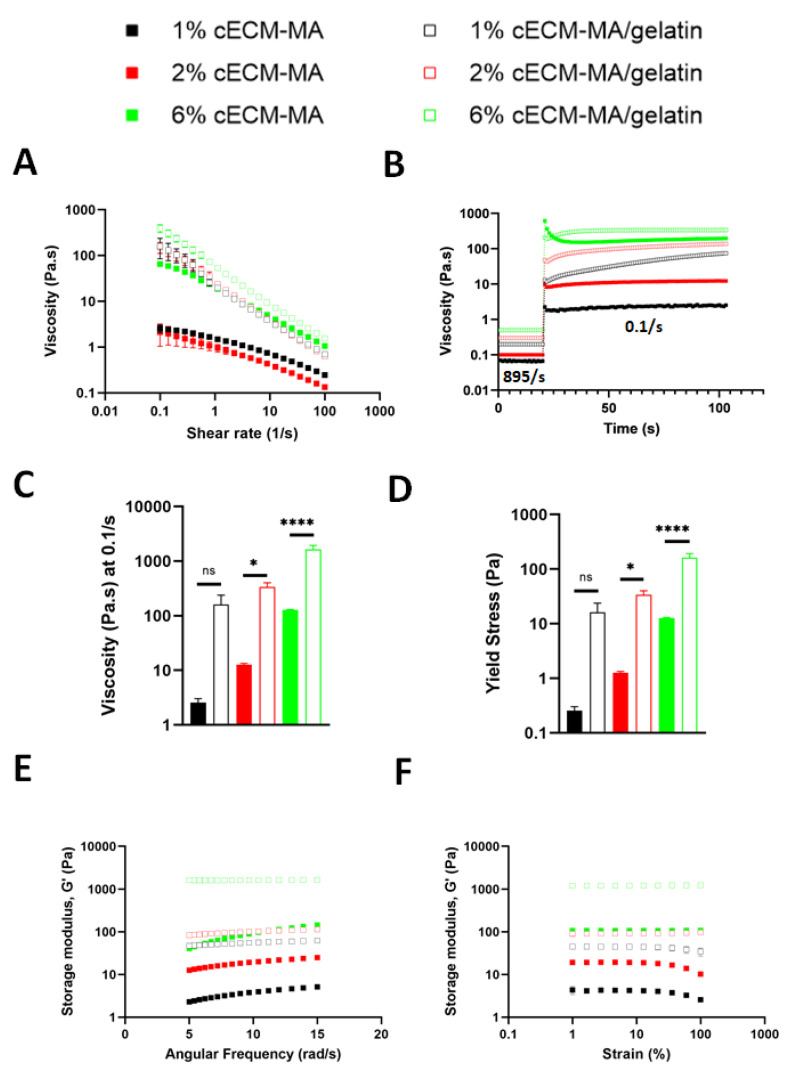
Rheological properties of formulations containing methacrylated cartilage ECM, with and without gelatin (*n* = 3). (**A**) Shear rate sweep from 0.1 to 100/s. (**B**) Shear recovery from 895/s (first 20 s) to 0.1/s (last 100 s). (**C**) Effect of inclusion of 3.5% gelatin on the viscosity of 1, 2, and 6% cECM-MA formulations at a shear rate of 0.1/s (close to zero shear) (ns = not significant; * = *p* < 0.05; **** = *p* < 0.0001). (**D**) Effect of the inclusion of gelatin on the yield stress (taken at 0.1/s) of 1, 2, and 6% cECM-MA formulations (ns = not significant; * = *p* < 0.05; **** = *p* < 0.0001). (**E**) Frequency sweeps of 1, 2, and 6% cECM formulation (only G’ shown for clarity). (**F**) Amplitude sweeps of 1, 2, and 6% cECM formulations (only G’ shown for clarity).

We next sought to characterise the rheological properties of 1, 2, and 6% cECM-MA. The decoration of ECM collagens with methacryloyl groups presents as an increase in both the storage and loss moduli (Figure S1). Furthermore, the viscosity at low shear rates is higher in the methacrylated ECM, but the viscosity at high shear rates converge for both the modified and unmodified ECMs (Figure S2). Recognising the need for higher viscosities to generate printable inks from the lower concentration cECMs, type A gelatin was added at 3.5% (*w/v*) as a temporary viscosity enhancer (unmodified gelatin will typically diffuse out of the structure in the hours following photocrosslinking of the cECM-MA). All materials (with and without gelatin) displayed shear-thinning behaviour under stress (Figure 3A). This was more pronounced for the bioinks supplemented with gelatin. Gelatin behaves as a Newtonian fluid (flow index = 1) at body temperature, and, upon cooling, random coils in the solution begin to interact and form physical crosslinks, whereby the gelatin undergoes a coil-to-helix transition and attains a gel state. This thermal behaviour of gelatin is key to forming a printable ink when it is added to the cECM-MA. The log values of the shear rate sweep data can be plotted with a linear fit to derive additional data related to the potential printability of the cECMs (Table 1). The slope of the line represents the flow index, n, which describes the type of shear response. Values < 1 indicate shear-thinning behaviour. The degree of shear thinning significantly increased when gelatin was added to the cECM, presenting as a decrease in the flow index. The consistency index, k, is the viscosity at a shear rate of 1/s. This value increases at higher cECM concentrations, suggesting better printability for formulations with high cECM content.

Another important aspect of printability is the recovery of the bioink post-shear. To assess this, shear recovery tests were undertaken, using 895/s as a typical shearing rate during extrusion [28]. In the gelatin-containing formulations, the viscosity continued to increase over time after the shear rate was reduced to 0.1/s (this temporal recovery was not observed in the gelatin-free formations (Figure 3B)). This indicates a re-establishment of the structure associated with the gelatin component at 13 °C. 

At a low “resting” shear rate (0.1/s), increasing the concentration of the cECM-MA from 2 to 6% resulted in a dramatic increase in viscosity (Figure 3C). The amount of stress required to initiate flow (known as the yield stress) is another parameter that determines shape retention post-extrusion. The addition of gelatin significantly increased the yield stress (Figure 3D) and the storage modulus (Figure 3E,F) for all formulations. 

The evolution of G′ and G″ during the photocrosslinking of 1% cECM-MA is shown in Figure S3. The elastic modulus of the formulation containing gelatin at 13 °C is higher than that without gelatin. This higher stiffness is due to at least partial gelation of the gelatin component at 13 °C. Irrespective of the gelatin component, the final value of G″ after photocrosslinking is the same for both formulations. At 2% cECM-MA, the same difference in G′ (due to the gelatin component) is apparent prior to the photocrosslinking step. However, the final G′ of the formulation without gelatin is higher after photocrosslinking (Figure S4), indicating that at higher cECM concentrations, the presence of gelatin may slightly retard the full crosslinking of all available sites within the cECM-MA component of the ink.

### 3.4. Rheological Properties and Printability of Cell-Laden cECM-MA Bioinks

**Figure 4:**
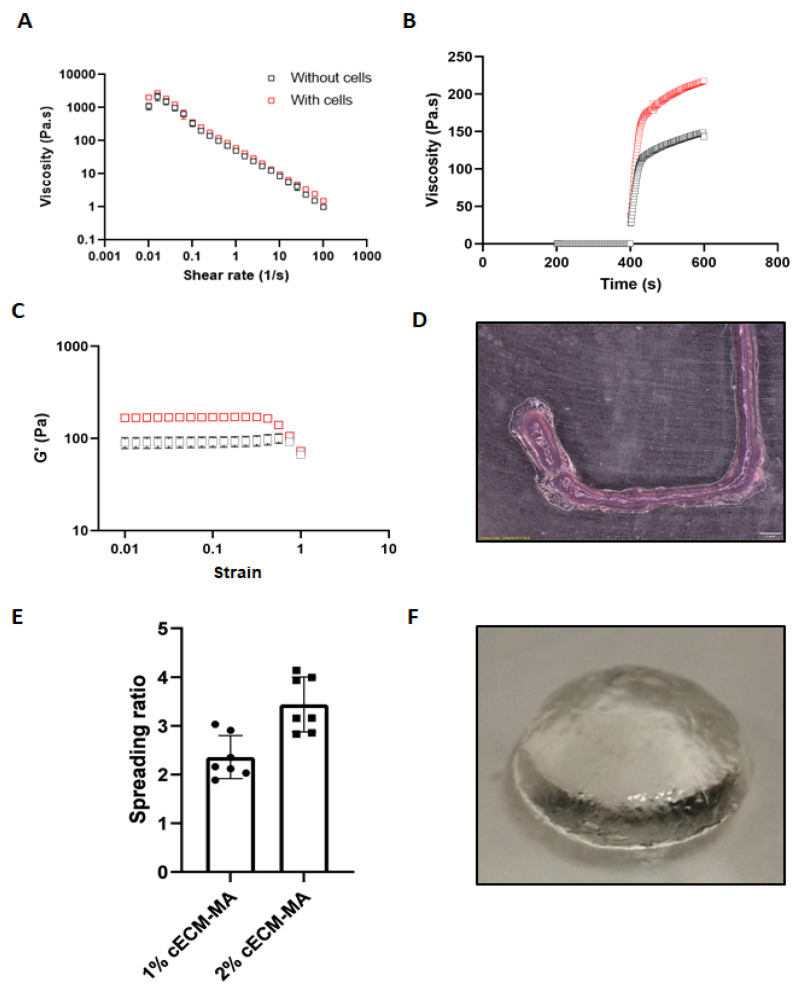
(**A**–**C**) Rheological properties of cell-laden, gelatin-supplemented cECM-MA bioinks versus acellular controls (*n* = 3). (**D**) Microextrusion of acellular 2% cECM-MA bioink. (**E**) Spreading ratio of acellular cECM-MA bioinks printed through 22G needle. (**F**) Printed acellular cylinder of 2% cECM-MA.

The 1 and 2% cECM-MA containing gelatin were next blended with BM-MSCs at a final density of 20 × 10^6^ cells/mL. The presence of cells in the formulation had no significant effect on the behaviour of the bioink, albeit slightly higher viscosities were observed for the cell-laden formulations. Both cell-laden and acellular formulations had almost identical shear-thinning behaviour (Figure 4A). However, the recovery of the cell-laden bioink post-shear was faster (Figure 4B). The presence of cells provided a higher storage modulus at low strains (Figure 4C).

To assess the printability of the bioinks, the spreading ratio post-printing was measured (Figure 4E). The spreading ratios of the 1 and 2% cECM-MA bioinks were 2.36 ± 0.34 and 2.70 ± 0.52, respectively. It was also possible to bioprint simple cylindrical shapes using the cECM-MA bioinks (Figure 4F). 

**Figure 5:**
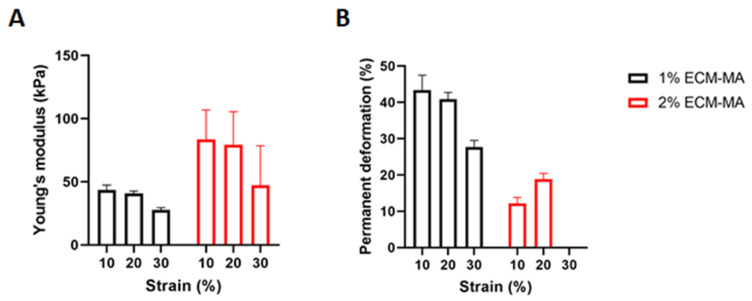
Compression testing of photocrosslinked cECM-MA hydrogels (*n* = 3). (**A**) Young’s modulus of compression (unconfined). (**B**) Permanent deformation due to compression.

Once printed, the constructs were exposed to UV light. The methacrylated collagens underwent radical polymerisation and self-crosslinked to form a gel. The mechanical properties of the cECM-MA hydrogels were then investigated using unconfined compression testing. The Young’s modulus increased with an increase in the concentration of the cECM-MA (Figure 5A). The higher concentration cECM-MA constructs also appeared to be more elastic, demonstrating lower levels of permanent deformation after the application of different strain levels (Figure 5B).

### 3.5. Chondrogenesis of MSCs in cECM-MA

**Figure 6:**
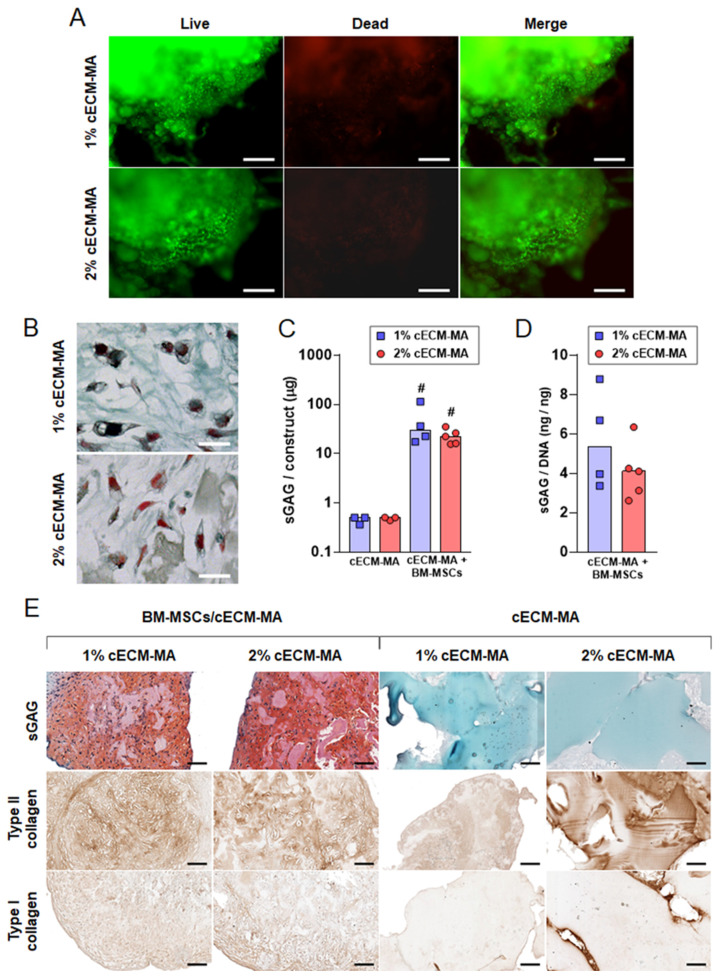
Biocompatibility and chondrogenic inductivity of cECM-MA. BM-MSCs were encapsulated in gels containing 1 or 2% cECM-MA and cultured in chondrogenic medium for 21 days in hypoxia. (**A**) Cell viability 7 days following encapsulation in cECM-MA. Living cells are stained in green, and dead cells are stained in red (scale bars = 200 µm). Histological (**B**,**E**) and biochemical (**C**,**D**) characterisations of tissue constructs after 21 days of in vitro culture. (**B**) Goldner’s Masson trichrome staining of cells encapsulated in cECM-MA (scale bars = 20 µm). (**C**) Quantification of the sGAG content of acellular gels or gels seeded with BM-MSCs. (**D**) sGAG content of cell-seeded gels normalised to DNA content. The values given in the dot plots represent individual scaffolds as data points with the median as bar; # indicates statistical significance compared to acellular gels (*p* < 0.05). (**E**) Representative pictures of parallel sections of constructs stained for type I and II collagens and sGAGs as indicated (scale bars = 100 µm).

To evaluate the capacity of the cECM-MA material to support chondrogenesis, BM-MSCs were encapsulated in 1 and 2% cECM-MA and cultured in a chondrogenic medium for 21 days. High levels of cell viability were observed after 7 days of culture in both cECM-MA concentrations (Figure 6A). After 21 days of chondrogenic induction in cECM-MA, the cells appeared to be round, displaying a morphology typical of chondrocytes (Figure 6B). Cells deposited sGAGs within the cECM-MA constructs over time in culture (Figure 6C,D), with no clear effect of the cECM-MA concentration observed. In agreement with these findings, safranin O/fast green staining further demonstrated a strong accumulation of sGAGs in the cell-seeded gels, whereas acellular gels did not stain for safranin O (Figure 6E). This observation is in line with the quantitative biochemical data indicating higher sGAG content in cell-seeded gels. Type II collagen staining increased with the concentration of the cECM-MA in acellular gels, which did not stain positive for type I collagen. Cell-seeded gels displayed a similar intensity of type II collagen-positive staining to acellular gels, whereas immunostaining for type I collagen appeared similarly weak in acellular and cell-seeded gels.

Together, these data indicate that the new material does not elicit a cytotoxic response at the concentrations tested. After encapsulation, the cells showed a round morphology within the cECM-MA gel, similar to what has previously been observed after the successful chondrogenic conversion of MSCs in collagen methacrylate hydrogels [21]. Thus, the cECM-MA gel allows intimate cell–scaffold interactions favourable for the differentiation of BM-MSCs into a chondrocyte-like cell. Positive staining for type II collagen and weak staining for type I collagen indicate that the material supports the production of hyaline-like cartilage rather than fibrocartilage. Taken together, our results demonstrate that the cECM-MA gel supports the chondrogenic differentiation of BM-MSCs and is suitable for cartilage bioprinting and tissue engineering applications.

## 4. Conclusions

Cartilage ECM was isolated, methacrylated, and formulated into a bioink. The concentration of the ECM in the formulations had measurable effects on the rheological properties. The printability of the cECM-MA bioink (and unmodified cECM at 6%) was demonstrated rheologically and by undertaking extrusion tests. The presence of cells in significant amounts (20 million/mL) had a negligible effect on bioink rheological properties. The cell-laden cECM-MA inks supported high levels of cell viability and robust chondrogenesis, demonstrating the applicability of these bioinks/hydrogels for 3D bioprinting and cartilage tissue engineering. 

## Figures and Tables

**Table 1:** Quantitative description of bioink rheological properties.

Bioink Formulation	Flow Index n	Consistency Index k
1% cECM-MA	0.66	1.45
1% cECM-MA + gelatin	0.29	20.8
2% cECM-MA	0.60	5.99
2% cECM-MA + gelatin	0.25	48.3
6% cECM-MA	0.38	19.3
6% cECM-MA + gelatin	0.18	60.3

## Data Availability

Data available upon request.

## Supplementary Materials

The following supporting information can be downloaded at: https://www.mdpi.com/article/10.3390/biom12020216/s1, Figure S1: Effect of methacrylation on the viscoelastic properties of ECM at 6% *w*/*v*. Figure S2: Effect of methacrylation on the viscosity of ECM at 6% *w*/*v*. Figure S3: Photo-rheology of 1% cECM-MA formulations (+/− gelatin). Figure S4: Photo-rheology of 2% cECM-MA formulations (+/− gelatin).
