# Supplementary material for: Methacrylated Cartilage ECM-Based Hydrogels as Injectables and Bioinks for Cartilage Tissue Engineering
Source: Biomolecules. 2022 Jan 27;12(2):216. doi: 10.3390/biom12020216 (PMC8961582; doi:10.3390/biom12020216)
Supplement: Supplementary file 1 [file biomolecules-12-00216-s001.zip › biomolecules-1526378-SI.pdf]

Supplementary Data

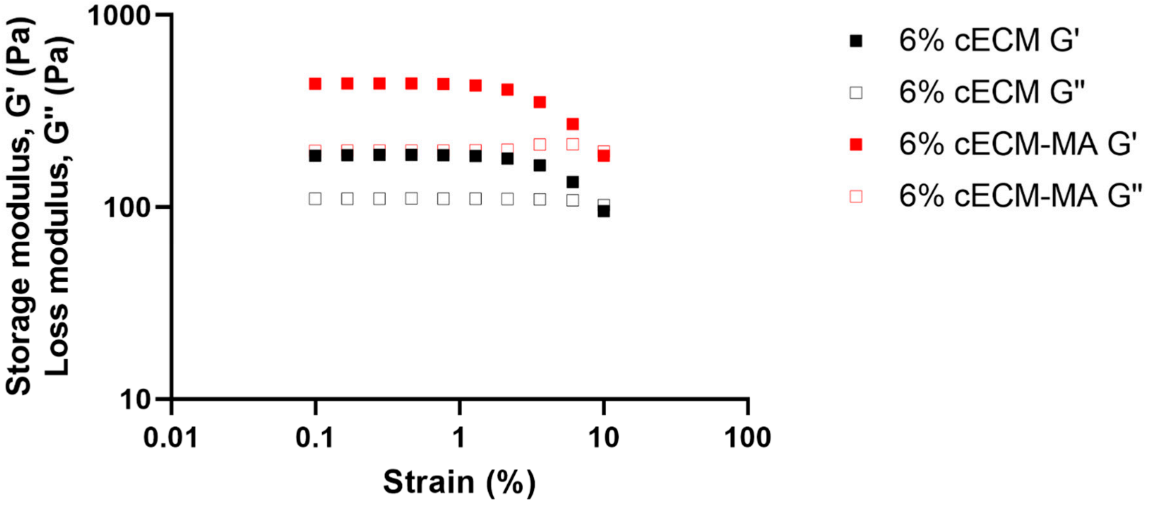

Figure S1: Effect of methacrylation on the viscoelastic properties of ECM at 6% w/v.

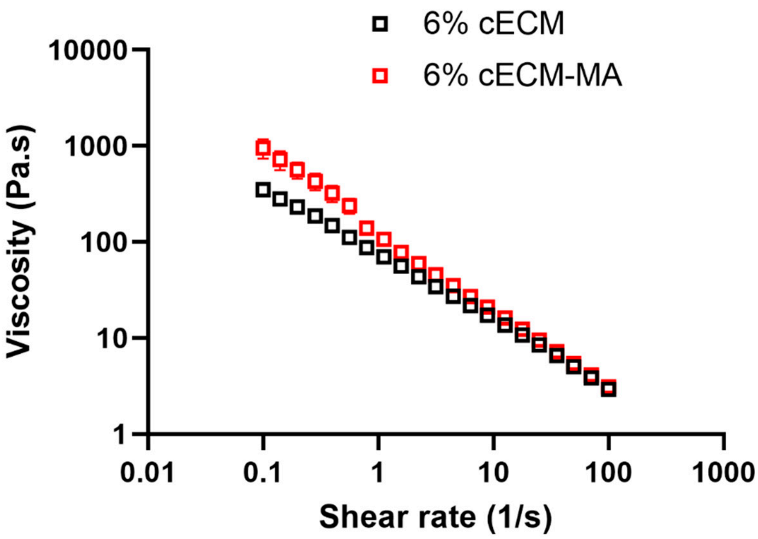

Figure S2: Effect of methacrylation on the viscosity of ECM at 6% w/v.

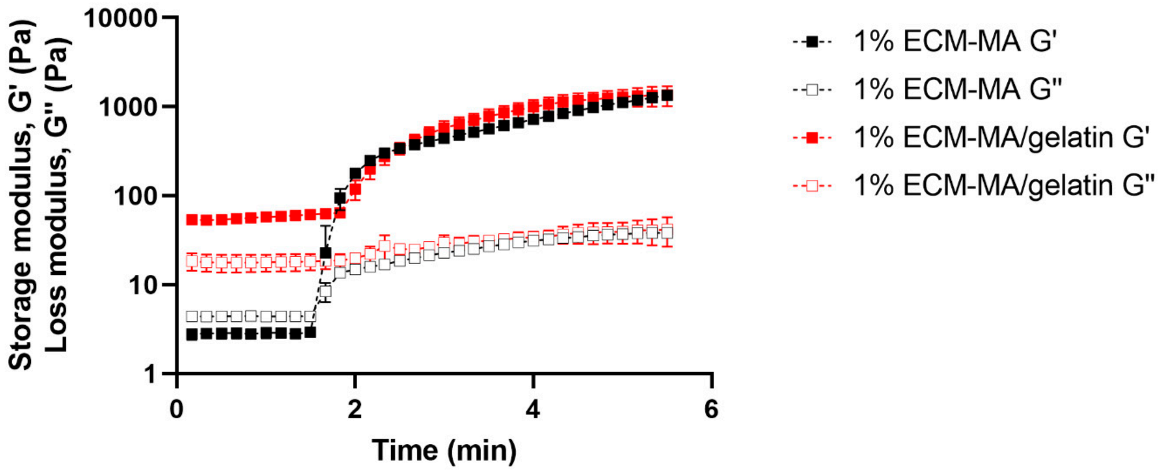

Figure S3: Photo-rheology of 1% cECM-MA formulations (+/- gelatin).

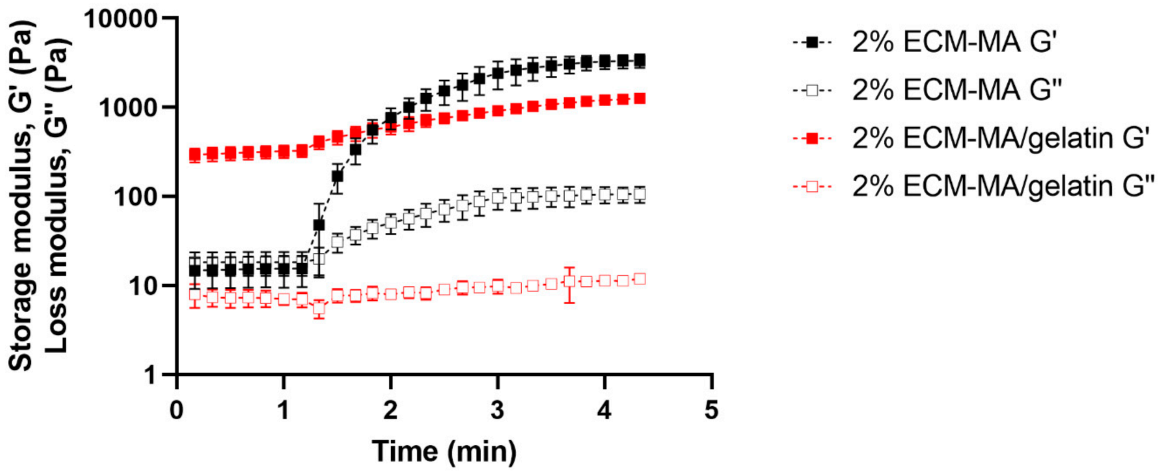

Figure S4: Photo-rheology of 2% cECM-MA formulations (+/- gelatin).
